# Space-time COVID-19 monitoring in Morocco

**DOI:** 10.11604/pamj.supp.2020.35.2.23505

**Published:** 2020-05-20

**Authors:** Fatine Hadrya, Abdelmajid Soulaymani, Faiçal El Hattimy

**Affiliations:** 1 Hassan First University of Settat, High Institute of Health Sciences, Unit of Epidemiology and Biomedical Sciences, Settat, Morocco; 2 Ibn Tofail University, Faculty of Sciences, Kenitra, Morocco

**Keywords:** COVID-19, space-time, distribution, control measures, Morocco

## Abstract

**Introduction:**

In Morocco, the first case was detected on March 02, 2020. Few days later, new cases are notified, followed by deaths. The government decided to adopt large-scale drastic measures against the epidemic. This study aims to describe and analyse the COVID-19 distribution in Morocco, according to key measures taken to curb this health problem.

**Methods:**

An observational study of all cases daily reported by the Ministry of Health of Morocco from 02 March to 05 May 2020, was carried out. A mapping was performed.

**Results:**

Before the implementation of the state of health emergency, 86 confirmed cases out of 471 biological examinations carried out have been reported and the highest cumulative incidence (0.43 cases per 100,000 inhabitants) was recorded in Fes-Meknes region. During the 1st quarantine, 2,960 positive cases out of 15,915 examinations carried out have been detected. Marrakech-Safi region was the most affected (15.33 cases per 100,000 inhabitants). Halfway through extended quarantine, 2,173 confirmed cases out of 49,570 biological examinations realized have been notified, the highest cumulative incidence has observed in Draa-Tafilalet region (27.45 cases per 100,000 inhabitants). The highest mortality was observed in Marrakech-Safi region (1.05 deaths per 100,000 inhabitants) and the highest case fatality rate (13.46%) was registered in Souss-Massa region.

**Conclusion:**

The strategy adopted by Morocco has enabled it to avoid a health catastrophe. But, it’s not over yet. Morocco should continue the massive efforts and reinforce the existing measures against the virus, especially in regions very affected by the epidemic.

## Introduction

In Wuhan, in Central China, a novel generation of SARS-CoV (SARS-CoV-2) has been isolated early December 2019 [1]. This virus spreads by respiratory droplets when patients cough, speak loudly or sneeze. Studies suggests that it may also be transmitted by contact between a contaminated hand and the mouth, nose or conjunctiva [2]. Common signs include respiratory symptoms, fever, cough, shortness of breath and breathing difficulties. In severe cases, it can engender pneumonia, severe acute respiratory syndrome, kidney failure and even death [3]. But despite a marked improvement in diagnostic response, a targeted therapies and vaccines are not yet available [4]. In January 2020, the number of cases has increased in China exponentially. The coronavirus disease 2019 (COVID-19) epidemic has spread across China and then reach many countries and territories [5]. Today, the problem has become global. We are talking about a pandemic, as declared by World Health Organization (WHO) on 11 March 2020, a real threat with the highest risk impact on all levels. The virus SARS-CoV-2 has crossed the Moroccan territory February 27, 2020.

The first case reported was a Moroccan resident returning from Italy. The 39-year-old patient had presented breathing difficulties, cough, cephalalgia and stomach-ache. In the hospital, the patient underwent a series of tests which turned out to be positive on 02 March 2020. He was placed in quarantine during 14 days. The people who were in contact with him were contacted and placed in enforced isolation and followed up daily [6]. Few days later, new positive cases appeared, followed by deceased cases. Morocco had the chance to analyse COVID-19 data early (since the onset of the pandemic worldwide) and decide to adopt large-scale drastic measures in accordance with the directives of his majesty the King Mohammed VI and under his direct supervision, in order to preserve the country. These measures have included the creation of a special fund to deal with the repercussions of this pandemic, the organization of the operation to return Moroccans from Wuhan [7], constraining mobility with a mandatory restrictive housing and curfew [8].

The state of health emergency in Morocco began with varying degrees of confinement, entered into force 20 March 2020, according to Legislative Decree No.2.20.293, to limit the spread of the disease from one individual to another and involved a novel medical protocol to “cure” infected patients with 2019-nCoV, according to Circular No.22 relating to the prescription and dispensation of Chloroquine and hydroxychloroquine at the level of healthcare establishments. In Morocco, numbers are communicated and percentages are calculated daily, as well as reports are written and disseminated to the general public. However, no serious epidemiological study has been developed to assess and analyse the health situation in Morocco as a whole, according to each principal measure of emergency. In this article, we will study and discuss the distribution of COVID-19 in Morocco through its spatial and temporal evolution, by referring to the key measures taken to manage the national health situation related to COVID-19.

## Methods

The data we used in this work are those published daily by the Ministry of Health of Morocco [9]. These data relate to the number of infected cases, deaths and recoveries since the appearance of the first imported case on 02 March 2020 until 05 May 2020. An observational study of all cases reported during this period was carried out.

**Definition of case and infection contact by 2019-nCoV:** possible case: anyone with an acute respiratory infection, fever and cough, as well as having travelled to China in the 14 days prior to onset of symptoms or anyone with an ARI, fever and cough within 14 days of close physical contact with a confirmed case of 2019-nCoV infection, or anyone who has worked or stayed in a hospital where a case of 2019-nCoV infection has been confirmed. In addition, any occurrence of grouped cases of serious acute respiratory infections hospitalized, with or without the concept of travel or residence in a hazardous geographic area, must be reported and investigated, in particular by nursing staff. Confirmed case: possible case with a sample indicating the presence of 2019-nCoV by molecular biology techniques, at a laboratory approved by the Ministry of Health. Excluded case: possible case with a sample indicating the absence of 2019-nCoV by molecular biology techniques, at a laboratory approved by the Ministry of Health. Infection contact: anyone who has been exposed to a possible or confirmed case, without appropriate protection; that is, a person who provided care to the patient (health professionals, close relatives); was in a situation of close and prolonged contact: (having lived with the case under the same roof, having had contact within one meter); has been in direct contact with respiratory secretions; has shared collective transport with the patent for an extended period.

**Reporting and notification of cases:** before any possible case, the health professional immediately reports the case to the delegation concerned of the Ministry of Health, by phone. Any possible case must be immediately notified by phone by the delegation to the regional directorates of health and the directorate of epidemiology and the fight against disease (National Centre of Operations of Public Health Emergencies Coordinator). The notification of the possible case and the contact of infection is made according to an established investigation form, accompanied by the list of contact persons of a confirmed or possible case of 2019-nCoV. As soon as a case is declared, an epidemiological investigation is undertaken by the Health Monitoring and Safety and Environmental Health Unit. These definitions are available in the report on the National Monitoring and Response Plan for 2019-nCoV infection prepared by the Moroccan Ministry of Health [10].

**Statistical analysis:** the data were exploited by Excel Office 2013. A brief description of the data is presented. The distribution of cases was assessed by the estimation of cumulative incidence, cumulative mortality and case fatality rate (CFR). To determine these rates, we used the population projection proposed by the directorate of the general census of population and housing of Morocco (the projection of 2020 from the census of 2014), reported by the high commission for planning [11]. Data mapping was carried out by ArcGIS 10.1 software.

## Results

On Tuesday 05 May 2020 at 4:00 p.m., the Moroccan Ministry of Health recorded 5,219 confirmed cases of infection, 1,838 recoveries (35.22%) and 181 deaths, with an average CFR of 3.47%. A total of 44,351 cases were excluded following a negative laboratory test result.

**Figure 1:**
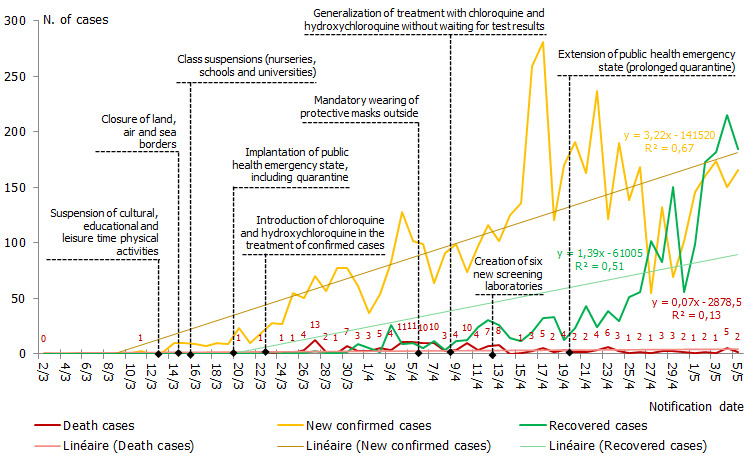
Temporal evolution of infected, recovered and deceased cases by COVID-19 in Morocco, until May 5^th^ 2020

**Temporal evolution of COVID-19:** in Figure 1, we can observe time evolution of COVID-19 epidemic in Morocco, until May 05, 2020. The first confirmed case was detected on Monday 02 March 2020 and few days later, other new cases was registered. Just after the first death notification, the Moroccan government decided to suspend all outdoor activities and classes as well as to close territorial borders in order to prevent the virus transmission. The state of public health emergency has been set up and quarantine has been imposed on Friday 20 March 2020. Thanks to measures taken early, the spread of the disease has been controlled. However, the number of recovered cases was very negligible. When Gautret and colleagues [12] publishes a study on therapy with chloroquine, Morocco adopts the treatment without delay. We are at war with an invisible enemy, so there is no time to lose. The treatment was directly applied to the confirmed serious cases. In parallel, the therapy was improved and then generalized in all health centres in the kingdom. As the number of cases continued to increase, wearing a mask became obligatory. At the start of the epidemic, COVID-19 screening was carried out only by two institutes.

Then, in order to increase the screening rate for the COVID-19 virus, the Ministry of Health opened other centres from the beginning of April 2020, in different regions of the country. Days before the end of quarantine (fixed on 20 April), the number of confirmed cases had increased; healthy carriers have started to develop the disease. Therefore, the quarantine was extended for 4 weeks. On average, in the past 2 weeks, the number of confirmed cases and deaths has halved, the number of cured cases has increased significantly. According to the regression analysis (trend line), the increase in the number of confirmed cases is highly dependent on time (explaining 67% of the evolution of the number of cases). This would be justified by the latency of the virus and the measures taken over time against its spread and a moderate association appears between the number of recovered cases and time (R2 = 0.51), most likely linked to the therapeutic protocol adopted. The results show that time has no relation to the death of infected cases.

**Figure 2:**
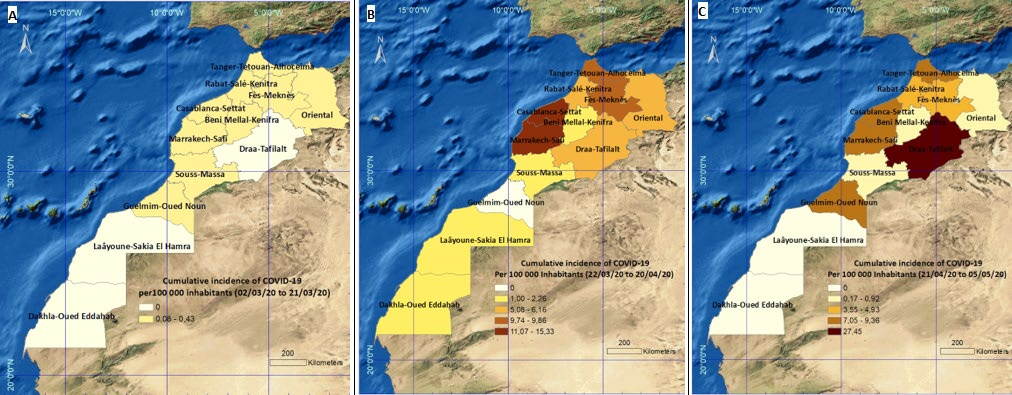
Regional distribution of cumulative incidence of COVID-19 cases per 100,000 inhabitants in Morocco. A) before implementation of the state of health emergency; B) during 1st phase of the state of health emergency; C) during 2^nd^ phase of the state of health emergency (until May 5^th^ 2020)

**Spatial evolution of morbidity by COVID-19:** before the implementation of the state of public health emergency, Morocco recorded 86 cases of COVID-19 out of a total of 471 biological examinations carried out (an average of 23.5 examinations per day). The detected cases were distributed in nine regions. The highest cumulative incidence (0.43 cases per 100,000 inhabitants) was recorded in Fes-Meknes region. No cases were detected in the east and south of the country (Figure 2 A). Then, the state of health emergency was established in the kingdom for the period March 21 - April 20, 2020 at first. This period was characterized by a significant growth in biological examinations performed (15,915 examinations) with a daily average about 530 biological examinations. These examinations made it possible to detect 2,960 cases distributed over the eleven of the twelve Moroccan regions. Marrakech-Safi region was the most affected with a cumulative incidence of 15.33 cases per 100,000 inhabitants, followed by Casablanca-Settat region with 11.07 cases per 100,000 inhabitants.

Three regions did not exceed 1.4 cases per 100,000 inhabitants; Laâyoune-Sakia El Hamra, Dakhla-Oued-Eddahab and Souss Massa. The cumulative incidence of Guelmim Oued Noun region went from 0.22 cases per 100,000 inhabitants (before the national entry into the state of health emergency) to 0 declared case (Figure 2 B). Given the increase in the number of infected cases just before the end of the quarantine period, the Moroccan government decided to extend the state of health emergency until May 20, 2020. The first two weeks were characterized by the appearance of several foci of family or professional contamination but also in closed places (penitentiary, barracks). Screening capacity has greatly increased, recording a total of 49,570 biological examinations, with an average of 3,304 examinations per day (until May 05, 2020). The country has recorded a total of 2,173 cases. The cumulative incidence has greatly increased in Draa-Tafilalet region, going from 5.08 cases (Figure 2 B) to 27.45 cases (Figure 2 C) per 100,000 inhabitants, becoming the highest rate since the beginning of the pandemic in this country.

This increase is mainly due to the cases registered (approximately 260 cases) in a local prison in the province of Ouarzazate. The cumulative incidence of regions like Casablanca-Settat, Tanger-Tetouan and Marrakech-Safi has remained stable, not exceeding 9.3 cases per 100,000 inhabitants. In contrast, the Guelmim Oued Noun region has once again become a reporting area (8.52 cases per 100,000 inhabitants), following the detection of a family foci in the city of Bouizakarne, located in the province of Guelmim. The cumulative incidence decreased slightly in three regions; Souss Massa, the Oriental and Beni Mellal-Kenitra). Two regions reported no case; Laâyoune-Sakia El Hamra and Dakhla-Oued-Eddahab (Figure 2 C). In spite of the prolongation of confinement, we note that the cumulative number of infected cases (stopped on May 5, 2020) tends more and more towards that determined during the 1st phase of quarantine, especially knowing that the number of infected cases per day varies between 100 and 200.

**Figure 3:**
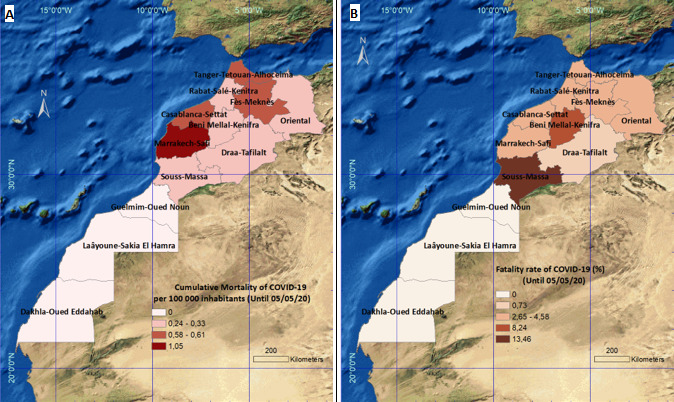
Regional distribution of mortality by COVID-19 in Morocco, until May 5^th^ 2020. A) cumulative mortality per 100,000 inhabitants; B) case fatality rate (%)

**Spatial evolution of mortality by COVID-19:** during the study period, the country recorded 181 deaths, with a mortality of 0.5 deaths per 100,000 inhabitants and a CFR of 3.47%. Figure 3 shows geographic distribution of mortality in Morocco through this period. The highest mortality was observed in Marrakech-Safi region (1.05 deaths per 100,000 inhabitants), followed by three regions; Casablanca-Settat, Fes-Meknes and Tanger-Tetouan-Al-Hoceima, with a mortality rate of around 0.6 deaths per 100,000 inhabitants in each of these regions, while the other regions had a rate lower than the national mortality rate recorded (Figure 3 A). As for lethality, Souss-Massa and Beni-Mellal-Khenifra regions recorded the highest rates; 13.46% and 8.24% respectively. It is suggested that serious cases have been mostly reported in these regions. The adjacent regions have reported rates ranging from 2.65 to 4.58%. Draa-Tafilalt region experienced lower CFR of 0.73% (Figure 3 B). The south of the country has not stated any deaths by COVID-19 (Figure 3).

## Discussion

The present study contributes to closely follow the evolution of COVID-19 in Morocco, after each measure taken by the Kingdom of Morocco to deal with the health repercussions of the spread of COVID-19. The main findings were that before the implementation of the state of health emergency, 86 confirmed cases out of 471 biological examinations carried out have been reported and the highest cumulative incidence was recorded in Fes-Meknes region. At the end of the 1st quarantine, 2,960 positive cases out of 15,915 examinations carried out have been detected, Marrakech-Safi region was the most affected. Halfway through extended quarantine, 2,173 confirmed cases out of 49,570 biological examinations realized have been notified, the highest cumulative incidence has observed in Draa-Tafilalet region. The highest mortality was observed in Marrakech-Safi region and the highest CFR was registered in Souss-Massa region.

International distribution (on a large scale): since COVID-19 spread more harshly in communities where there are mass gatherings [13], the virus is transmitted to other geographical regions through infected travellers quickly [14]. By the end of February, when Morocco has not yet declared a case of COVID-19 infection, China reached 80,000 infected cases including 2,838 deaths and further 53 countries notified approximately 6,000 confirmed cases, as well as 86 deaths [15]. To leave open the territorial borders while the disease began to spread inside China seems to be the first mistake. Morocco has detected the first COVID-19 case on March 02, 2020. Andorra, Indonesia, Jordan, Latvia, Portugal, Saudi Arabia, Senegal and Tunisia have identified their first case the same day [16]. As of 21 March 2020, a total of 292,142 cases were reported worldwide in 173 countries with 12,783 deaths. China has notified most infection cases in the world (81,498 cases including 3,267 deaths) whereas Italy reported most deaths outside China; 4,827 deaths out of 53,578 infected cases.

After Italy and China, the countries where the most people died are Iran with 1,556 deaths (20,610 cases), Spain with 1,326 deaths (24,926 cases) and France with 562 deaths (14,296 cases). However, according to the data in Table 1, the highest CFR was in Algeria, of 15.96%, which still undervalued. Morocco has recorded 86 infected cases and 3 deaths (CFR = 3.49%) on this date. In some countries, late taken seriously the epidemic was devastating (Table 1). The COVID-19 ended up wreaking havoc on all levels (e.g. health, economy, society), thereby negatively affecting patient outcomes as well as the population. It’s the case of China (from January 25, 2020, with the peak of the Chinese spring festival holidays) [17], Spain (celebration of numerous mass demonstrations authorized on 08 March (Women´s day), mainly in the city of Madrid, as well as many mass celebrations in the Fallas of Valencia and sports celebrations) [18] and Iran (from April 9, 2020, some 66,220 confirmed cases and 4,110 deaths, coincided with Nowruz (the Persian New Year) holidays) [19]. During celebrations and holidays, highest annual human movement patterns around the country and/or through the world have observed, increasing the potential for rapid geographic spread of the illness [19].

**Table 1:** COVID-19 situation in twenty countries, according to the periods marking Morocco; before quarantine, during quarantine, during prolonged quarantine

	Overview until May 5, 2020			Before quarantine in Morocco			During quarantine in Morocco (1st phase)			During quarantine in Morocco (2nd phase)		
Countries	Confirmed cases	Death cases	CFR (%)	Confirmed cases	Death cases	CFR (%)	Confirmed cases	Death cases	CFR (%)	Confirmed cases	Death cases	CFR (%)
USA	1,171,185	62,698	5.35	15,219 a	201 a	1.32 a	736,054 a	35,683 a	4.85 a	419,912	26,814	6.39
Spain	219,329	25,613	11.68	24,926	1,326	5.32	175,284	19,526	11.14	19,119	4,761	24.90
Italy	213,013	29,315	13.76	53,578	4,827	9.01	127,650	19,287	15.11	31,785	5,201	16.36
UK	194,994	29,427	15.09	5,018	233	4.64	119,729	16,276	13.59	70,247	12,918	18.39
Germany	164,897	6,996	4.24	21,463	67	0.31	121,994	4,531	3.71	21,440	2,398	11.18
France	131,292	25,491	19.41	14,296	562	3.93	99,217	19,671	19.83	17,779	5,258	29.57
Brazil	107,780	7,321	6.79	904	11	1.22	37,750	2,451	6.49	69,126	4,859	7.03
Iran	99,970	6,340	6.34	20,610	1,556	7.55	62,895	3,653	5.81	16,465	1,131	6.87
China	84,406	4,643	5.50	81,498	3,267	4.01	2,752	1,375	49.96	156	1	0.64
S. Arabia	30,251	200	0.66	392	-	-	10,092	103	1.02	19,767	97	0.49
Portugal	25,702	1,074	4.18	1,280	12	0.94	19,583	723	3.69	4,839	339	7.01
Mexico	24,905	2,271	9.12	164 a	1 a	0.61 a	8,097 a	685 a	8.46 a	16,644	1,585	9.52
Ireland	21,983	1,339	6.09	785	3	0.38	14,867	684	4.60	6,331	652	10.30
S. Korea	10,806	255	2.36	8,897	104	1.17	1,786	133	7.45	123	18	14.63
S. Africa	7,439	148	1.99	240	-	-	3,060	58	1.89	4,139	90	2.17
Egypt	7,201	452	6.28	285	8	2.81	3,048	242	7.94	3,868	202	5.22
Malaysia	6,383	106	1.66	1,183	3	0.25	4,241	86	2.03	959	17	1.77
Morocco	5,219	181	3.47	86	3	3.49	2,960	140	4.73	2,173	38	1.75
Algeria	4,838	470	9.71	94 a	15 a	15.96 a	2,624 a	369 a	14.06 a	2,120	86	4.06
Thailand	2,989	55	1.84	411	1	0.24	2,400	47	1.96	178	7	3.93
Tunisia	1,022	43	4.21	60	1	1.67	824	37	4.49	138	5	3.62

* Numbers include both domestic and repatriated cases a Underestimated numbers (one day since last reported case) USA: United States of America; UK: The United Kingdom; S. Arabia: Saudi Arabia

Thus, these countries have immediately adopted a state of health emergency, closing all borders, and applying strict protection measures. More than 900 million people in more than 35 countries around the world are finally called to stay at home by the authorities. Most of these nations are subject to obligatory confinement; like Italy [20], Spain [18], Iran [21], India [22] and Brazil [15]. The rest are subject to a curfew, quarantine or non-coercive calls not to leave their homes, such as Japan [23] and South Korea [24]. In Morocco, the response to the COVID-19 was immediate: it occurred prior to the first cases being discovered in Morocco. For hold back the infection by the 2019-nCoV, the government has prepared and implemented a national monitoring and response plan to the disease. To effectively fight this serious disease, he took more stringent measures to significantly limit travel instead of simply advising citizens to stay at home. Such as Morocco and Brazil [25], few countries have studied the extent of the problem early and have stated health emergency against the COVID-19 in time.

During 4-week of quarantine of Morocco, three countries have known sharp increases of new cases each day (i.e. Italy, Spain and the United States) because either delayed or lacked sufficient access to reliable testing for many reasons. Italy, Spain and the United States chose to falsely reassure the populace and to downplay the threat of COVID-19. The pandemic had grown out of control in those countries and had overwhelmed healthcare facilities. As a result, Italy, Spain and the United States are among the many countries experiencing severe social and economic turmoil today [26]. In Morocco, the reported new cases and deaths have grown but remained below other countries with almost similar conditions (Table 1). Surprisingly, once the date of the end of confinement approached, new foci of contamination appeared, obliging the authorities to extend the state of health emergency by 4 weeks and to apply sanctions for not wearing a protective mask. In these 2 weeks, the overall health situation has improved, inside and outside Morocco, even if in some countries, the CFR still high (Table 1). The Table 1 summarizes the COVID-19 situation in twenty countries [16], according to the periods marking Morocco; before quarantine, during quarantine, during prolonged quarantine. We can see the trend of the COVID-19 pandemic. Note that the reported cases are those with non-mild symptoms requiring hospitalization.

National distribution (on a small scale): the pandemic has affected the whole country, with incidence rates varying from region to region. These variations might partly be related to the density of the urban population which constitutes a risk factor for the spread of this pandemic in Africa [27]. Undeniably, the first reporting areas involved cities with a large demography such as Casablanca city (Casablanca-Settat region), Marrakech city (Marrakech-Safi region) as well as Fez and Meknes cities (region of Fes-Meknes) [11]. Besides, like most epidemics, the fast spread of COVID-19 will disproportionately affect the most disadvantaged people. Professional sources of contamination (nearly 400 cases in factories and shops in Marrakech, Fez, Casablanca and Tangier) and in penal establishments in Ouarzazate (Draa-Tafilalt region), Ksar El Kebir (Tangier-Tetouan region) and Loudaya (Marrakech-Safi region) have also emerged and put these regions in front of others in terms of morbidity. Prisons are epicentres for infections because of the high-risk multimorbidity and the poor access to healthcare services relative to that in community settings [28].

These infections can be transmitted between prisoners and staff [29]. Additionally, inadequate investment in prison health, substantial overcrowding in some prison settings, and rigid security processes have the potential to delay diagnosis and treatment [30]. That is why strict corrective and preventive measures have been taken against this problem in Morocco after these episodes, as well as daily monitoring of infection contacts, to prevent a new outbreak occurs in this kind of places. As for mortality, variations between regions may be linked to the old age and the comorbidities. In Morocco, age advancement is more and more accompanied by an increase in the prevalence of chronic diseases [31,32]. In addition, studies have shown that this virus causes a higher mortality rate in older adults and those with comorbidities such as hypertension, cardiovascular disease, diabetes, chronic respiratory disease and chronic kidney disease [3,33,34]. The likelihood of having multiple comorbidities places older adults at an even greater risk of increased mortality from SARS-CoV-2 [17,33,35]. Thus, early vigilant monitoring along with high quality supportive care are needed in patients at high risk [36].

Overall, the CFR has dropped considerably, ranging from 3.49 and 4.73, to 1.75, which is a significant achievement. The considerable decrease in CFR indicates a resumption of control of the health situation of the Moroccan population; an improvement in the quality of care and the specific conditions to which patients are subject. The cumulative number of recovered cases is constantly increasing, justified in particular by the new therapeutic protocol to which patients are subjected. As in China [37], South Korea [24] and Brazil [25], the provision of information and communication to the population (on measures to distance individuals, isolation of cases and reminders of good hygiene practices) was adopted as a fundamental strategy for addressing the epidemic in Morocco. Efforts have been directed towards reinforcing health surveillance and health care, as well as boosting research, development and innovation.

## Conclusion

Morocco has learned a lot from the experiences of countries severely affected by the COVID-19 pandemic. It was therefore better equipped to reach satisfactory results and better control the health situation of the Moroccan population. We are on the right path. The fight is not over. Definitely, the strategy adopted by Morocco has enabled it to avoid a health catastrophe, but the country is still “at war” against the spread of the virus in the various regions of the kingdom. Today, one region and several provinces are declared without any active COVID-19 cases, but three scenarios can arise: we are ready to lift the confinement on 20 May 2020 by proceeding with a progressive deconfinement; it´s is necessary to extend again the confinement; we can coexist with the virus, adopting basic prevention measures. Morocco should continue the massive efforts and reinforce the existing measures against the virus. This assessment of the COVID-19 epidemic needs to be modified as and when new information is added. However, the need is stressed for the scientific community and national and international epidemiological surveillance teams to take great care when monitoring the epidemic´s trends, critically assessing the tools available for understanding the situation.

### What is known about this topic

- Raw data (numbers), some graphics and maps are available to the general public on COVID-19 and its impact on Morocco;
- Only non-periodic government reports and daily press articles fairly concise and relating to specific aspects (political, economic, etc.), are published on their respective websites.

### What this study adds

- This study gives a global vision then detailed (by period) of the health situation of Morocco related to COVID-19;
- This study shows the strengths and weaknesses of the measures taken to counteract the disease;
- This study allows to compare the evolution of COVID-19 in Morocco with that of some twenty countries.

## Competing interests

The author declares no competing interests.
